# In Vivo Retroviral Transduction of Cardiac Myofibroblasts Using Intramyocardial Injection Immediately Post-myocardial Infarction

**DOI:** 10.21769/BioProtoc.5500

**Published:** 2025-11-20

**Authors:** Satsuki Ono, Hayato Watanabe, Yuma Horii, Michio Nakaya

**Affiliations:** 1 Graduate School of Pharmaceutical Sciences, Kyushu University, Fukuoka, Japan; 2 Department of Disease Control/Research Institute of Environmental Medicine, Nagoya University, Aichi, Japan

**Keywords:** Intramyocardial injection, Myocardial infarction, Retroviral transduction, Myofibroblast, Fibrosis

## Abstract

Following myocardial infarction (MI), myocardial cells undergo cell death, and the necrotic region is replaced by extracellular matrix (ECM) proteins such as collagens. Myofibroblasts are responsible for producing these ECM proteins. Cardiac myofibroblasts are differentiated from resident fibroblasts in response to inflammation. To date, genetically modified mice driven by the Periostin promoter and adeno-associated virus 9 (AAV9) carrying the Periostin promoter have been used for gene transfer into cardiac myofibroblasts. However, these methods require multiple steps and are time-consuming and expensive. Therefore, we developed a method for delivering genes into cardiac myofibroblasts using retroviruses. Specifically, the DNA of the target gene was transfected into Plat-E cells, which are packaging cells, to generate retroviruses. The virus-containing supernatant was then harvested, and the viruses were pelleted by centrifugation and suspended in PBS-containing polybrene. Subsequently, permanent occlusion of the left coronary artery was performed, and 20 μL of viral solution was immediately administered using a 29G needle at a position 1–2 mm below the ligation site in the heart of mice maintained in an open chest state. Using this method, we were able to introduce genes into the myofibroblasts of interest surrounding the MI site.

Key features

• Retroviruses are taken up only by proliferating cells, enabling highly specific gene transfer into myofibroblasts.

• Any gene incorporated into the genome by retroviruses will continue to be expressed over the long term, providing chronic in vivo evaluation.

• Myocardial injection targeting the infarct area of the left ventricle shows high infection efficiency in myofibroblasts.

• This protocol employs a very small-scale and simple virus concentration method.

## Graphical overview


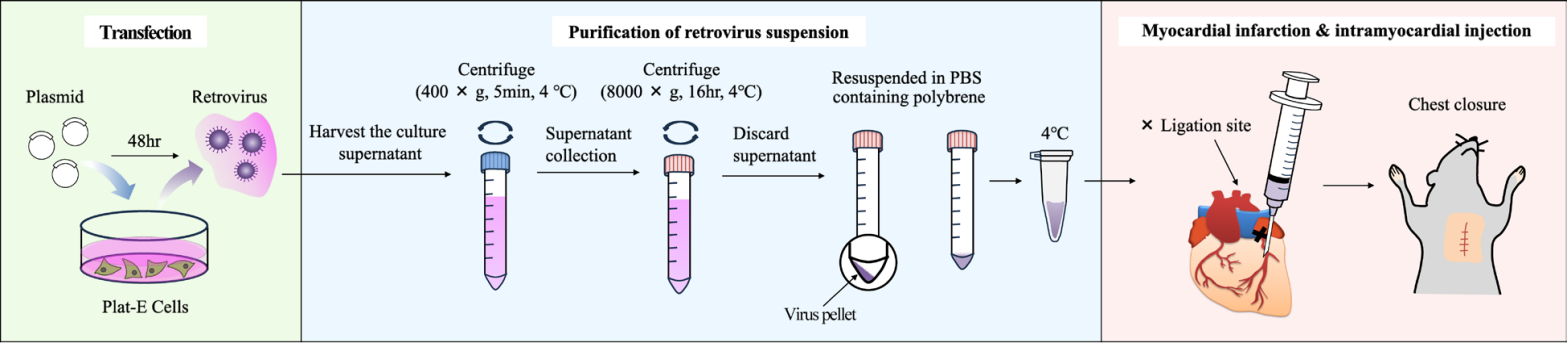


## Background

To date, gene delivery technology to cardiac myofibroblasts has been performed by administering AAV9 or AAV-DJ constructs containing Cre recombinase driven by the promoter of Periostin, a marker gene for myofibroblasts, via the tail vein [1,2]. In addition, transgenic mice using the promoter of Periostin have been created as a method of introducing genes into cardiac myofibroblasts [3,4].

Keeping genetically modified mice is time-consuming and expensive. This protocol does not employ a promoter-driven strategy and does not require mouse breeding. The purification of AAV and other viruses requires multiple steps and an ultracentrifuge, which is expensive. Our protocol utilizes a relatively inexpensive floor-standing centrifuge and completes the concentration of the virus solution in a single centrifugation step. Therefore, this procedure can be readily performed by most researchers.

Considering that myofibroblasts in the fibrotic area have extremely high proliferative capacity and that retroviruses are readily incorporated into dividing cells, we considered that administering retroviruses to the heart after myocardial infarction (MI) treatment might be effective to introduce genes into myofibroblasts. Other cell types, such as endothelial cells, leukocytes, and pericytes, are also present in the fibrotic region, and it was assumed that these cells might also proliferate; fortunately, no genes were introduced into these cells. This is expected to be due to the lack of expression of the receptors required for retroviral infection in these cells.

We found that the protein Vestigial-Like Family Member 3 (VGLL3) is specifically expressed in cardiac myofibroblasts after MI [5]. Interestingly, experiments using isolated cardiac myofibroblasts revealed that VGLL3 translocated from the cytoplasm to the nucleus depending on mechanical stimulus. To verify this in vivo using intramyocardial injection immediately post-MI, EGFP-VGLL3 was expressed only in myofibroblasts in the fibrotic region, where the tissue stiffness is increased by the accumulation of collagens and localized to the nucleus. These results indicate that VGLL3 is a mechanosensitive protein in myofibroblasts in fibrotic hearts.

This protocol is expected to be applied in various fields of research on heart diseases involving cardiac myofibroblasts. Below, we introduce the retroviral transduction protocol of cardiac myofibroblasts using intramyocardial injection immediately post-MI.

## Materials and reagents

**Biological materials**

1. Plat-E cells; cells containing *gag, pol*, and *env* genes, allowing retroviral packaging with a single plasmid transfection were provided by Dr. Kitamura, University of Tokyo, Japan. Plat-E cells are also commercially available (Cell Biolabs, catalog number: RV-101)

2. Wild-type house mouse (*Mus musculus*) (Japan SLC, catalog number: C57BL/6JmsSlc)

**Reagents**

1. Serum-free DMEM (Nacalai Tesque, catalog number: 08458-16)

2. FBS (Gibco, catalog number: 10270-106)

3. Penicillin-streptomycin (Nacalai Tesque, catalog number: 09367-34)

4. PEI-Max (Polysciences, catalog number: 24765-1)

5. Water deionized and sterilized (Nacalai Tesque, catalog number: 06442-95)

6. NaOH (Nacalai Tesque, catalog number: 31511-05)

7. Polybrene (Sigma-Aldrich, catalog number: 107689-10G)

8. Phosphate buffered saline (PBS) (Nacalai Tesque, catalog number: 14249-95)

9. Evans Blue (WAKO, catalog number: 056-04061)

10. EGFP-VGLL3/pMXs-puro [constructed in our laboratory; pMXs-puro retroviral vector (Cell Biolabs, catalog number: RTV-012)]

**Solutions**

1. Culture medium (see Recipes)

2. PEI Max solution (see Recipes)

3. Plasmid solution (see Recipes)

**Recipes**

**1. Culture medium**

**Table:** 

Reagent	Final concentration	Volume (mL)
Serum-free DMEM	89% (v/v)	445
FBS (inactivation)	10% (v/v)	50
Penicillin-streptomycin	1% (v/v)	5
Total	n/a	500

Prior to addition, inactivate FBS by heating at 56 °C for 30 min in a water bath. Then, filter it with a 0.2 μm filter.

Store the culture medium at 4 °C. Stable for at least one month.

**2. PEI Max solution**

**Table:** 

Reagent	Final concentration	Quantity or volume
Water deionized and sterilized	n/a	40 mL
PEI-Max	1 μg/μL	0.04 g
Total	n/a	40 mL

After mixing the reagents, adjust the pH to 7.4 with NaOH. Store the solution at 4 °C. Stable for at least 6 months.

**3. Plasmid solution**

**Table:** 

Reagent	Final concentration	Volume (μL)
Serum-free DMEM	94% (v/v)	564
Plasmid (EGFP-VGLL3/pMXs-puro)	10 μg/mL	6
PEI Max solution (Recipe 2)	50 ng/mL	30
Total	n/a	600

Prepare just before use. Use high-quality plasmid DNA (e.g., purified using a cesium chloride gradient or an endotoxin-free maxiprep kit) at a concentration of 1 μg/μL.

**Laboratory supplies**

1. Pipette tips, 1,000 μL (Thermo Fisher Scientific, Thermo Scientific^TM^, catalog number: 2179-HR)

2. Pipette tips, 200 μL (Thermo Fisher Scientific, Thermo Scientific^TM^, catalog number: 2069-HR)

3. Pipette tips, 20 μL (Thermo Fisher Scientific, Thermo Scientific^TM^, catalog number: 2149P-HR)

4. Pipette tips, 10 μL (Thermo Fisher Scientific, Thermo Scientific^TM^, catalog number: 2140-HR)

5. Filter units (Thermo Fisher Scientific, Thermo Scientific^TM^, catalog number: 569-0020)

6. 10-cm cell culture dish (Nippon Genetics, catalog number: FG-2090)

7. 15 mL tube (Thermo Fisher Scientific, Thermo Scientific^TM^, catalog number: 339650)

8. 15 mL tube (AS ONE Corporation, catalog number: 1-3500-01)

9. Surgical tape (3M, catalog number: 1527-0)

10. Razor blade (FEATHER, catalog number: FH-10)

11. 8-0 braided silk (NATSUME SEISAKUSHO, catalog number: M6-80B2)

12. 5-0 braided silk (NATSUME SEISAKUSHO, catalog number: ER12-50B1)

13. 29G 0.5 mL syringe (TERUMO, catalog number: SS-10M2913)

14. 1.7 mL tube (BM Bio, catalog number: BM-15)

## Equipment

1. Pipetman 1,000 μL (Gilson, catalog number: F123602)

2. Pipetman 200 μL (Gilson, catalog number: F123601)

3. Pipetman 20 μL (Gilson, catalog number: F123600)

4. Aspirator (TOKYO RIKAKIKAI, catalog number: A1000S)

5. Respirator (Shinano Manufacturing, catalog number: SN-480-7X2T)

6. Heating pad (Marukan, catalog number: RH-207)

7. Optical microscope (Olympus, catalog number: SZX7)

8. Tweezer (NATSUME SEISAKUSHO, catalog number: A-12-2)

9. Tweezer (MEISTER, catalog number: 2-AXAL)

10. Small scissors (NATSUME SEISAKUSHO, catalog number: B-12)

11. Forceps (NATSUME SEISAKUSHO, catalog number: C-1)

12. Clean bench (Panasonic Healthcare, catalog number: MCV-B131S)

13. Water bath (TAITEC, catalog number: Personal-11)

14. Centrifuge (TOMY SEIKO, catalog number: LC-200)

15. Centrifuge (TOMY SEIKO, catalog number: AX-321)

16. CO_2_ incubator (SANYO, catalog number: MCO-18AIC)

## Procedure

**A. Generation of retrovirus preparations (days 1–5)**

**Day 1**

1. Seed 2.5 × 10^6^ Plat-E cells into 10-cm dishes with 10 mL of culture media (see Recipes).

2. Incubate in a 5% CO_2_ incubator at 37 °C overnight.

**Day 2**

3. Add 600 μL of plasmid solution (see Recipes) dropwise to the culture medium using a Pipetman.

4. Incubate in a 5% CO_2_ incubator at 37 °C for 48 h.

**Day 4**

5. Collect 10 mL of supernatant from the culture medium of Plat-E cells at 90%–100% confluency into a 15 mL tube.

6. Centrifuge at 400× *g* for 5 min at 4 °C using centrifuge (LC-200).

7. Collect 9 mL of the supernatant into a new 15 mL tube (AS ONE).

8. Centrifuge the collected retrovirus suspension at 8,000× *g* for 16 h at 4 °C using centrifuge (AX-321) (see General note 1).

**Day 5**

9. Discard the supernatant using an aspirator and a Pipetman.

10. Suspend the virus pellet in 40 μL of PBS-containing polybrene (4 μg/mL) and transfer to a 1.7 mL tube (tube 1).

*Note: If you want to visualize whether the retroviral suspension has been successfully administered to the myocardium, add Evans Blue so that the final concentration is 0.1%–1%.*

11. Keep the retrovirus suspension (tube 1) in an ice bath (4 °C) until just before use.

*Note: To minimize the loss of viral titer associated with freeze–thaw cycles, we recommend using freshly prepared retrovirus. The suspended virus can be stored in an ice bath (4 °C) for 12 h without the loss of viral titer.*

**B. Intracardiac injection immediately post-MI (day 5)**

**Day 5**

1. Prepare an 8–10-week-old male wild-type mouse of C57BL/6J strain.

*Note: Male mice are chosen to be subjected to the MI operation to avoid the risk of acquiring variable data due to the estrous cycle of female mice.*

**Figure 1.**
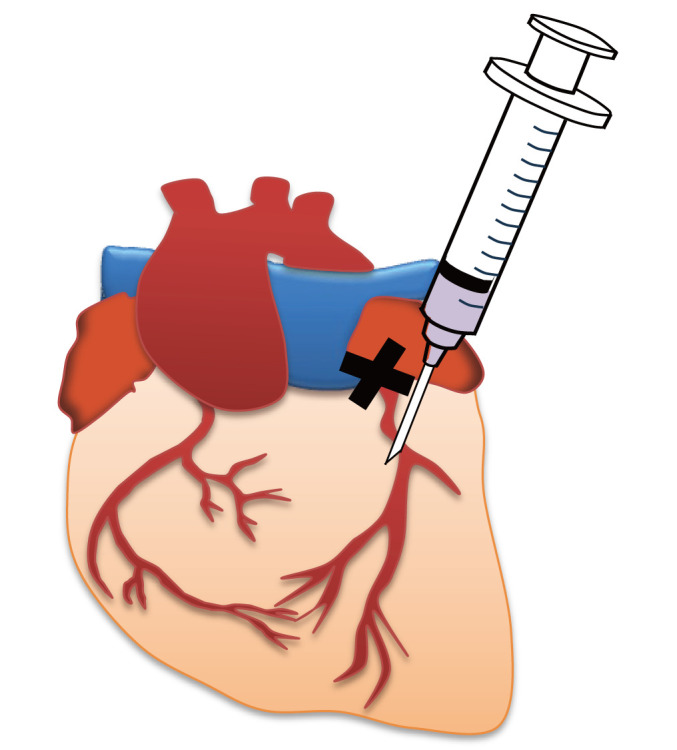
Image of myocardial administration immediately after myocardial infarction (MI). The X indicates the ligation site.

2. Generate a myocardial infarction model by ligating the descending coronary artery according to Maruyama et al. [6].

3. Using a 0.5 mL syringe, administer 20 μL of the retroviral suspension prepared in section A (tube 1) into the myocardium 1–2 mm below the ligation site (see Figure 1 and Video 1).

*Note: Insert the tip of the needle at an angle of 5–15° from the base of the heart toward the apex and until the bevel of the needle is buried so that the virus suspension does not leak out.*

**Critical:** Do not reinsert the syringe needle and make more than two holes, as this may cause arrhythmia and increase the mortality rate.

**Video 1.**
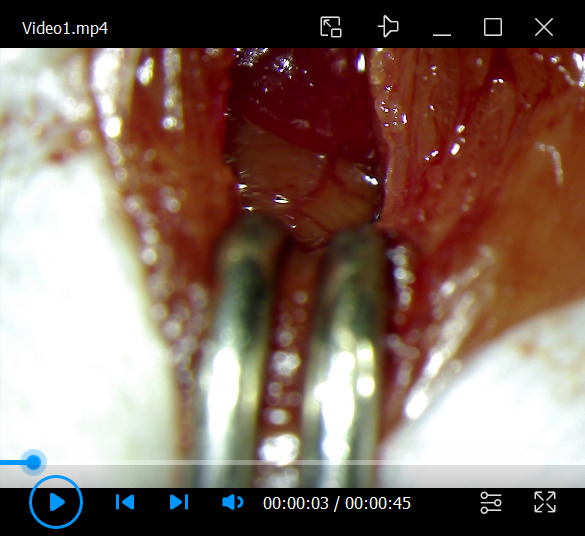
How to administer myocardial injection immediately after myocardial infarction (MI) establishment. Insert the tip of the needle at an angle of 5–15° from the base of the heart toward the apex, 1–2 mm below the ligation site (black thread: 8-0 braided silk). In this video, the retroviral suspension was stained with Evans Blue.

4. Close the chest using 5-0 braided silk.

5. Perform artificial respiration with a respirator for at least 30 min (volume of air for respiration: 0.5 mL, respiratory frequency: 120 bpm).

6. After the operation, place the mouse prone into the cage on a heating pad until recovery from anesthesia.

## Data analysis

**Figure 2.**
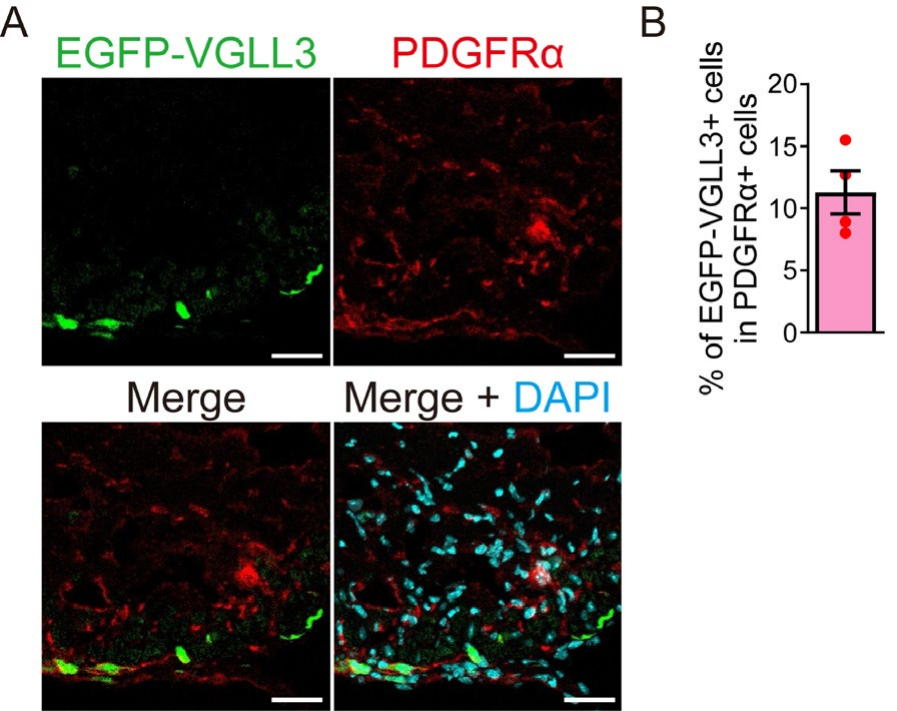
Quantification of retroviral transduction efficiency. (A) Signals for EGFP-VGLL3, PDGFRα, and DAPI in the left ventricle of myocardial infarction (MI) murine hearts on day 3.(B) Percentage of EGFP-VGLL3-positive cells in PDGFRα-positive cells. The number of PDGFRα-positive cells is 60–80 per field (n = 4). The graph represents the means ± SEM. Scale bar = 20 μm.

The efficiency of retroviral transduction was assessed by quantifying PDGFRα-positive myofibroblasts expressing EGFP-VGLL3 using Blue Zen 2012 (Zeiss). PDGFRα was used as a myofibroblast marker in this analysis because αSMA (the most representative myofibroblast marker) expression is weak in the cardiac myofibroblasts 3 days after myocardial infarction (Figure 2A). The proportion of EGFP-VGLL3-positive cells among PDGFRα-positive cells was 11.3% (Figure 2B).

## Validation of protocol

**Figure 3.**
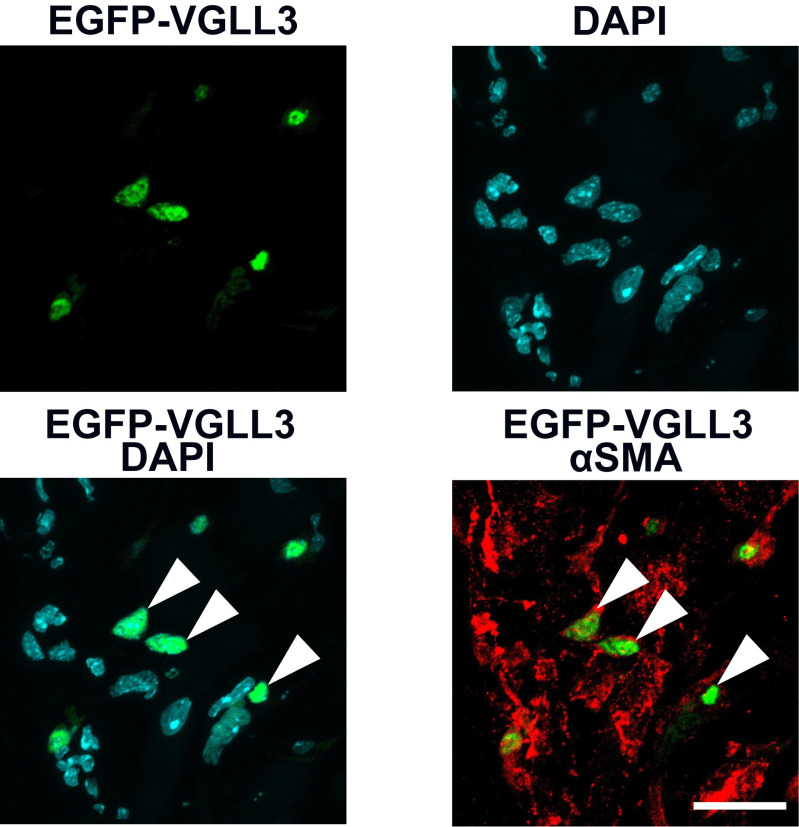
Nuclear localization of EGFP-VGLL3 in myofibroblasts in the left ventricle of myocardial infarction (MI) murine hearts on day 3. This figure is reused from Horii et al. [5]. The intracellular localization of EGFP-VGLL3 in cardiac myofibroblasts was assessed using immunohistochemistry. Myofibroblasts were labeled by staining with αSMA, a major marker. The white arrowheads in the merged image indicate representative signals of VGLL3 in the nucleus of cardiac myofibroblasts. The mouse heart was fixed in 4% PFA/0.1 M phosphate buffer overnight. Scale bar = 20 μm.

This protocol has been used and validated (Figure 3) in the following research article: Horii et al. [5] VGLL3 is a mechanosensitive protein that promotes cardiac fibrosis through liquid–liquid phase separation. *Nature Communicati*ons (Figure 1k).

**Figure 4.**
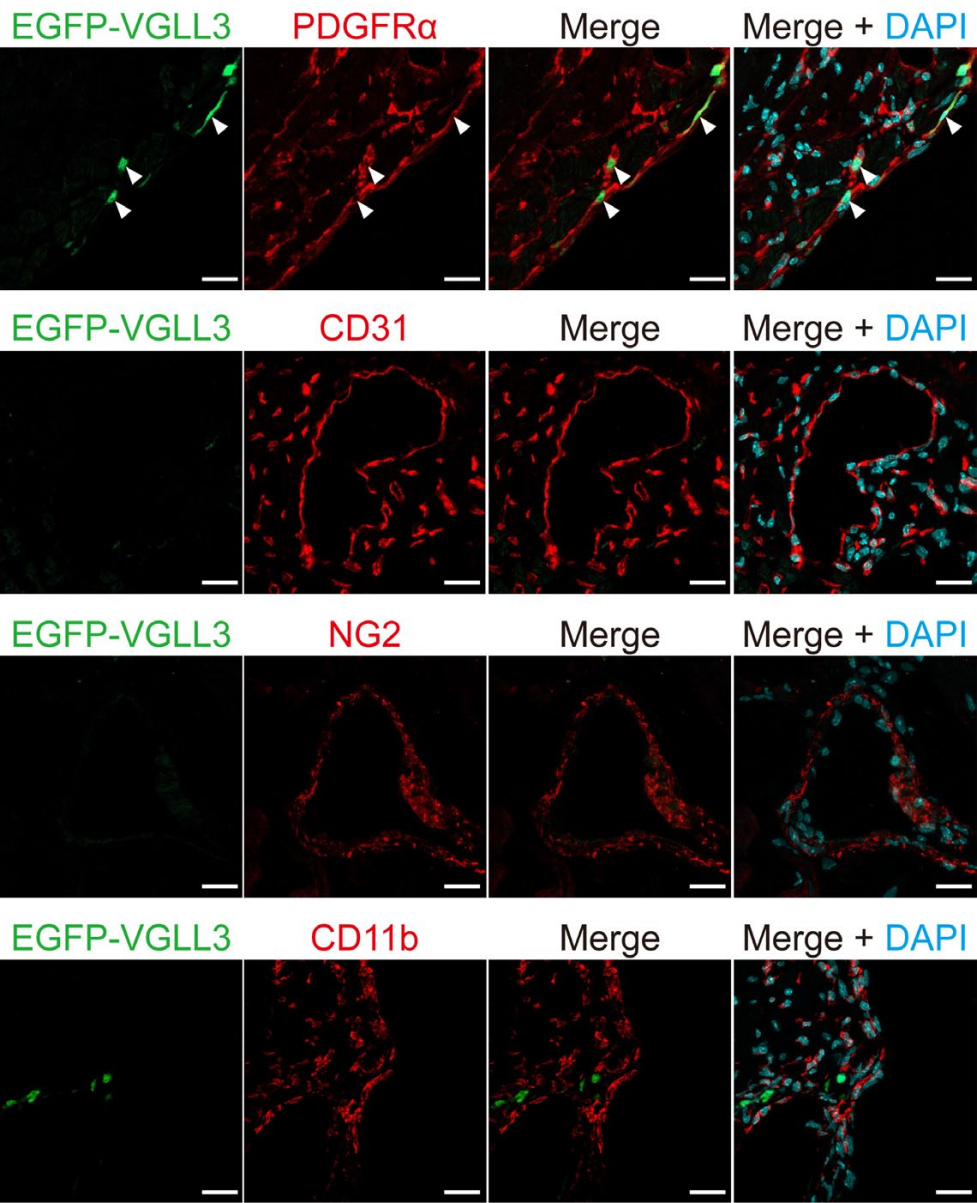
EGFP-VGLL3 was not expressed in endothelial cells, leukocytes, or pericytes in the left ventricle of infarcted mouse hearts three days after injection of retrovirus. Representative images in immunohistochemistry assessed for EGFP-VGLL3, PDGFRα (a fibroblast marker), CD31 (an endothelial cell marker), CD11b (a leukocyte marker), and NG2 (a pericyte marker). The white arrows in the images indicate representative signals of EGFP-VGLL3. The mouse heart was fixed in 4% PFA/0.1 M phosphate buffer overnight. Scale bar = 20 μm.

Our immunohistochemical analysis revealed that EGFP-VGLL3 is retrovirally expressed specifically in cells that are positive for PDGFRα and is not expressed in endothelial cells (CD31-positive cells), leukocytes (CD11b cells), and pericytes (NG2-positive cells) (Figure 4).

## General notes and troubleshooting

**General notes**

**Figure 5.**
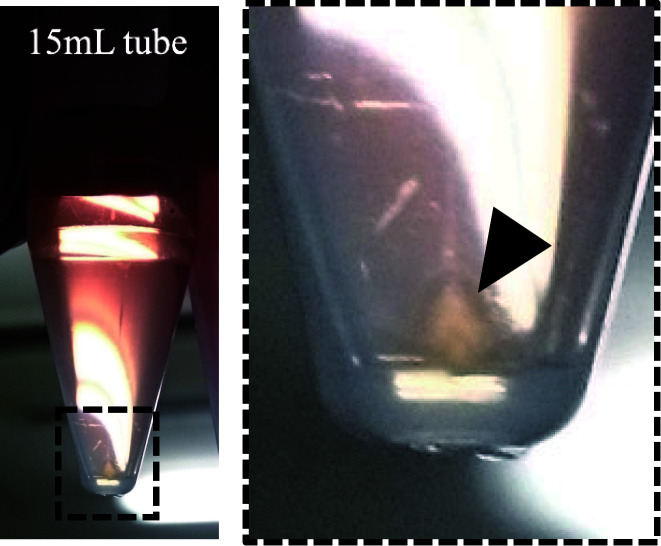
An image of the retrovirus pellet observed after centrifugation. The black arrowhead in the image indicates the retrovirus pellet.

1. After centrifugation for 16 h, the retrovirus pellet can be seen at the bottom of the tube (see Figure 5) (see step A8).

2. In European countries, the generation of retrovirus vectors requires an S2 safety laboratory and special legal requirements.

3. To prepare the retrovirus suspension, viruses should be collected and centrifuged one day before myocardial infarction and intramyocardial injection.

**Troubleshooting**

**Problem 1:** Most mice die after MI treatment followed by intramyocardial injection.

Possible cause: The mouse's chest was open for a long time.

Solution: In intramyocardial injection immediately after MI, it is desirable to complete the procedure from opening to closing the chest within 30–40 min.

**Problem 2:** Mouse dies immediately after injection.

Possible cause: The needle penetrated the ventricle lumen, injecting the virus into the bloodstream or causing fatal arrhythmia.

Solution: Ensure the needle is inserted at the shallow recommended angle and that the bevel is fully within the tissue before injecting.

**Problem 3:** Low retrovirus infection efficiency.

Possible causes: The retrovirus titer could be low. The virus supernatant was not fresh (harvested >48–72 h post-transfection). The depth of the needle insertion is inappropriate.

Solution: Increase the amount of supernatant collected from Plat-E cells and centrifuge to increase the viral titer. Always harvest supernatant at 48 h precisely and concentrate immediately. Insert the needle until the bevel of the needle is buried so that the virus suspension does not leak out. At the same time, do not insert the needle too deeply so that it does not penetrate the left ventricle.

**Problem 4:** No virus pellet is visible after centrifugation.

Possible causes: Transfection efficiency was low; insufficient virus was produced.

Solution: Check transfection efficiency on Plat-E cells (e.g., by co-transfecting with a fluorescent plasmid). Increase the number of dishes used for virus production.
